# Does effective gaze behavior lead to enhanced performance in a complex error-detection cockpit task?

**DOI:** 10.1371/journal.pone.0207439

**Published:** 2018-11-21

**Authors:** Stephanie Brams, Ignace T. C. Hooge, Gal Ziv, Siska Dauwe, Ken Evens, Tony De Wolf, Oron Levin, Johan Wagemans, Werner F. Helsen

**Affiliations:** 1 Movement Control & Neuroplasticity Research Group, Department of Kinesiology, KU Leuven, Leuven, Belgium; 2 Experimental Psychology, Department of Psychology, Helmholtz Instituut, Utrecht University, Utrecht, The Netherlands; 3 The Academic College at Wingate, Wingate institute, Netanya, Israel; 4 CAE Oxford Aviation Academy, Brussels, Belgium; 5 Laboratory of Experimental Psychology, Department of Brain & Cognition, KU Leuven, Leuven, Belgium; University of Tübingen, GERMANY

## Abstract

The purpose of the current study was to examine the relationship between expertise, performance, and gaze behavior in a complex error-detection cockpit task. Twenty-four pilots and 26 non-pilots viewed video-clips from a pilot’s viewpoint and were asked to detect malfunctions in the cockpit instrument panel. Compared to non-pilots, pilots detected more malfunctioning instruments, had shorter dwell times on the instruments, made more transitions, visited task-relevant areas more often, and dwelled longer on the areas between the instruments. These results provide evidence for three theories that explain underlying processes for expert performance: The long-term working memory theory, the information-reduction hypothesis, and the holistic model of image perception. In addition, the results for generic attentional skills indicated a higher capability to switch between global and local information processing in pilots compared to non-pilots. Taken together, the results suggest that gaze behavior as well as other generic skills may provide important information concerning underlying processes that can explain successful performance during flight in expert pilots.

## Introduction

The relationship between perceptual-cognitive skills and human expert performance has been of interest in various domains (e.g., sports [1, 2], surgery [3], driving [4]). Based on the cognitive information processing approach [5], such skills can be defined as the ability to process environmental information, compare it to internal representations of the external world, and use the information to organize and produce movements that are relevant for a successful completion of a task. Among other things, perceptual-cognitive skills can lead to better anticipation and decision-making through several underlying processes, one of which is a more efficient use of vision to extract relevant environmental information [6].

The efficient use of vision and the ability to comprehend visual information while integrating other sensory experiences relevant to a successful completion of a task plays an important role in superior performance. It is also well-known that experts in various domains rely on vision to accomplish their tasks, whether they are surgeons, athletes, drivers, or police officers, and that experienced performers or experts may use vision differently than less experienced individuals or near-experts. Hence, the specific “gaze behavior”–the purposeful use of the visual system to extract relevant information from the environment in order to produce an optimal action associated with a specific task–is expected to differ between experts and less-expert performers or between high and low performing individuals (within a specific domain of expertise). Indeed, such differences were reported in aviation [7, 8], police shooting [9], sports [10, 11] and surgery [12].

Gaze behavior consists of several types of eye movements. Fixations, for example, keep the gaze stable on environmental stimuli, while saccades are rapid eye movements, typically from one fixation location to another [13]. Hence, variables such as the number and duration of fixations or amplitudes of saccades are expected to differ between expert and less expert performers. In this respect, a recent meta-analysis of Gegenfurtner and colleagues [14] suggested three theories that explain the role of gaze behavior as a mechanism of perceptual-cognitive expertise in expert performance: (i) the long-term working memory theory [15], (ii) the information-reduction hypothesis [16], and (iii) the holistic model of image perception [17].

The *long-term working memory theory* suggests that experts encode and retrieve information from memory more rapidly than their less experienced counterparts. According to this theory, long-term working memory can be considered an extended part of the working memory [15]. Based on this theory, the faster and more efficient information processing of experts should lead to shorter fixation durations, or dwell times (i.e., the accumulated duration of fixations in a specific area of interest), since it is assumed that experts are able to extract more meaningful information from those shorter fixations or dwells [14]. Evidence for the long-term working memory theory was observed in a wide range of expertise domains: (i) In radiology, shorter dwell times on nodules were reported in experts for successful diagnoses [18]; (ii) in sports, expert judges in rhythmic gymnastics were reported to use shorter fixation durations on the scene for error-detection in the performance of the gymnasts [19], and (iii) in the military, experts were reported to use shorter fixation durations during scene evaluation for risk assessment [20].

The *information-reduction hypothesis* suggests that experts are better at selectively attending to task-relevant information and ignoring task-irrelevant information [16]. Based on this hypothesis, it is expected that experts will make less fixations of shorter duration to task-irrelevant areas and more fixations of longer durations to task-relevant areas [14]. This theory is also supported by studies on expert’s gaze behavior in different domains of expertise. For example: (i) expert pediatrics scanned less over the whole scene but fixated more on relevant areas in videos of children with seizures before setting a correct diagnosis [21]; (ii) expert football referees fixated more on the most informative area of the attacking player (contact zone) and spend less time fixating the body part that was not involved in the infringement (non-contact zone) during the assessment of foul play situations [22], and (iii) highly experienced tennis players focused more on relevant body parts of the opponent player while less experienced players fixated on the racket, leading to a better anticipation in a temporal occlusion tennis task [23].

The *holistic model of image perception* suggests that experts have an extended visual span. This allows them to first globally analyze a scene by using their parafoveal vision, and based on this analysis, direct their attention to relevant areas [17]. Based on this theory, it is expected that experts will have longer saccade amplitudes to cover more areas and will take less time to fixate on task-relevant areas [14]. Again, previous research addressing differences in gaze behavior between experts with different experience levels provided evidence for this theory. For example: (i) expert cardiologists were faster to fixate on relevant sections of the electrocardiogram (ECG) and used longer foveation time on the QRS complex during interpretation of the ECG plot [24]; (ii) during the diagnosis of histopathological plaques in a virtual microscope, experts fixated faster on the diagnostic relevant area [25], and (iii) expert radiologists appeared to fixate faster on fractures during analyses of skeletal radiographs [26].

The eye-movement features that have been proposed to characterize expertise differences in gaze behavior according to the three theories are not mutually exclusive and can be complementary [14]. In fact, expert performers can adapt their gaze strategies across different specific situations in order to achieve their objective. In Gegenfurtner and colleagues’ meta-analysis, 92 effect sizes supported the long-term working memory theory, 69 effect sizes supported the information-reduction hypothesis, and 29 effect sizes supported the holistic model of image perception. These results suggest that the gaze behavior of expert performers can vary. Variations in gaze behavior could partly be explained by task characteristics. Nonetheless, evidence from this meta-analysis suggests that gaze behavior is strongly affected by the level of expertise within specific expertise domains. In this respect, one would expect to find significant differences in gaze behavior strategies between participants with different levels of expertise and/or performance differences within a variety of expertise domains [24, 27–29]. Indeed, evidence for a flexible search behavior in experts has been presented in various sport-related domains: soccer players [30]; baseball players [31]; or soccer referees [22]. For example, skilled players change their search rate according to the distance from their opponents [30] or had the capacity to rely on peripheral visual information when the central vision no longer supported task performance [31]. Elite soccer referees spend more time fixating on contact zone and less time on non-contact zone of the attacking player in open play situations, whereas differences in the visual search behavior between elite and sub-elite referees were less evident in corner kick situations [22].

Besides eye-movement events that characterize gaze behavior (i.e., fixations and saccades), also specific visual scanning patterns have been identified as a predictor of expert performance across multiple domains of expertise. In radiology, for example, systematic scanning patterns were linked to higher detection rates of lung lesions whereas unstructured scanning patterns have been shown to account for about 30% of the missed lung lesions in RX-thorax analysis [28, 31]. Moreover, it has been shown that adapting systematic scan pattern through training can reduce miss-detection errors and improved diagnostic performance among radiologists [32]. Previous results reported clear differences in visual scanning between expertise groups during analyses of electrocardiograms, namely a more systematic scan pattern was observed in the expert group, while the students used random scanning [24]. This suggests that visual scan patterns would likely affect expert performance and can be used to differentiate between high and low performing individuals or decide about the level of expertise [24, 27–29, 33–36]. A measure to indicate the systematicity of a scan pattern is the scan entropy. The lower this value, the more systematic the used scan pattern [37].

One domain where optimal gaze behavior and systematic scan patterns are essential for performance of complex human tasks in which performers are required to react correctly to unpredicted events and make complex decisions under stress is aviation [36, 37]. While there are many eye-tracking studies in aviation [37], there are almost no studies that examined pilots’ gaze behavior and performance in relation to the three proposed theories. For example, in Gegenfurtner and colleagues’ meta-analysis only three out of 73 reviewed studies were conducted in pilots [38–40]. In one of those studies [39], expert pilots made more fixations of shorter durations compared to novice pilots during a landing approach in a flight simulator. These results are in line with the long-term working memory theory. However, in another study [40], compared to less expert pilots, expert pilots spent more time gazing at task-relevant areas and less time gazing at task-irrelevant areas. These results are in line with the information-reduction hypothesis. Also, systematicity in the scan pattern has been shown to be important in-flight performance [37]. Based on previous literature, it appears that, at least in aviation, systematic visual scan patterns are also related to improved perceptual-cognitive performance [36, 38, 39, 41, 42]. A measure that can be used to assess systematicity of scan patterns is visual scanning entropy [36, 43]. The lower the entropy, the more systematic the visual scan pattern is.

Piloting an aircraft requires the simultaneous performance of various tasks. Usually, continuous tasks (e.g., flying the aircraft) need to be performed simultaneously with serial tasks (e.g., following a pre-landing checklist) and discrete tasks (e.g., answering the air traffic controller, changing a radio frequency). In addition, in modern commercial aircraft equipped with modern automated technologies, pilots spend more time monitoring instruments rather than physically flying the aircraft [37, 44]. Hence, being able to detect changes in the state of the aircraft, and specifically, being able to detect malfunctions (e.g., instruments that give wrong indications, stop functioning, or indicate a malfunction of the aircraft itself) is of importance. Recently, a concern was raised that ineffective monitoring of aircraft instruments was a contributing factor in many flight accidents [45]. One tragic example was the 2009 crash of Colgan Air flight 3407. One of the contributing factors to this crash was “the flight crew’s failure to monitor airspeed in relation to the rising position of the low-speed cue” [46]. Unfortunately, in this crash, two pilots, two flight attendants, and 45 passengers perished. In another accident in 2009 –Air France flight 447 –due to discrepancies in airspeed instruments, some autopilot systems disconnected. The crew failed to identify deviations from flight path and a stall condition (i.e., a condition in which the aircraft loses lift and can no longer be flown) leading to a tragic crash in the Atlantic Ocean that killed three flight-crew members, nine cabin-crew members, and 216 passengers (French Civil Aviation Safety Investigation Authority, 2012).

Clearly, pilots’ ability to monitor the aircraft’s instruments is of the utmost importance. This ability is expected to be related to superior perceptual-cognitive skills in addition to a high ability to process information from multiple sources. Specifically, in the current study, we analyzed gaze behavior according to the three theories which are associated with superior perceptual-cognitive skills as well as scan entropy and other attentional generic skills as described below. By using eye-tracking measurements, our aim was two-fold. First, to characterize the relationship between gaze behavior, scan entropy, and performance during completion of an error-detection cockpit task between different expertise and performance groups and second, to explain the findings with the proposed three theories of gaze behavior in experts; namely, the theory of long-term working memory [15], the information-reduction hypothesis [16], and the holistic model of image perception [17].

### Gaze behavior and scanning entropy as indicators for expertise and superior perceptual-cognitive skills in aviation

Gaze behavior and scanning entropy can be used as indicators for expertise and superior perceptual cognitive skills in aviation. Therefore, in the current study, eye movements were recorded during completion of a task where participants were instructed to detect a malfunctioning cockpit instrument during simulated flight, the error-detection cockpit task. In line with the notion that the three above-mentioned theories are complementary rather than exclusive, we hypothesize that, compared to non-pilots: (1) pilots will make more transitions and use shorter dwell times (in line with the long-term working memory theory), (2) pilots will make fewer dwells of shorter durations to task-irrelevant areas and more dwells of longer durations to task-relevant areas (in line with the information-reduction hypothesis), and (3) pilots will have a wider visual span and a shorter time to first dwell on the failing instrument after the error occurred (in line with the holistic model of image perception). Taking into account previous findings suggesting a more systematic scan pattern in experts compared to non-experts, which is indicated by a lower scan entropy value, we further hypothesize that (4) pilots’ scan entropy will be lower compared to non-pilots. Finally, since, previous research results indicated inter-individual differences in perceptual-cognitive skills between pilots [47], these hypotheses will also be checked for differences between high and low performers within the same expertise group (see the Materials and methods section for details). (5) High performing participants (irrespective of their level of expertise) are expected to adapt an expert-like gaze behavior which might combine specific eye-movement features that will not be evident in the low performing pilot group or in the non-pilot group (irrespective of their performance level).

### Generic attentional skills as indicators for expertise and superior perceptual-cognitive skills in aviation

Beside gaze behavior, generic attentional skills are expected to be another process underpinning perceptual-cognitive skills in pilots. Specifically, we argue that differences in mental workload between pilots with different levels of experience could affect their ability to detect malfunctioning cockpit instruments and that individual differences in mental workload are related to generic attentional skills [48]. Moreover, previous research reported a relation between visual scanning and workload in pilots [34, 49]. In this study, the rated perceived exertion (RPE [50]) was used to assess differences in workload between expertise and performance groups. Results of a previous study showed that increased mental workload tends to increase the dwell time [51–53]. According to the above-mentioned hypotheses, an increased dwell time is expected in the non-pilot group. In line with these previous results, we hypothesize that (6) non-pilots and/or low-performers show an increased workload for task completion compared to the pilot and/or high performing group. Based on the knowledge that mental workload and generic attentional skills are related, a domain-general approach, which examines the relationship between domain-specific expertise and domain-generic perceptual-cognitive skill, was added in this study. Previous research results reported significant relationships between the performances in domain-generic perceptual-cognitive skill measures and the performance in the domain-specific task [3, 54–57]. In contrary, other research results reported no relation between domain-generic and domain-specific skills [58]. To our knowledge, until now, no research was conducted assessing the relationship between domain-generic and domain-specific skills in pilots. Although, during complex cockpit tasks there is a need for extraordinary attentional skills, and the capability to capture essential information from multiple inputs. Since these might be underlying generic skills that might explain superior performance in pilots, two generic tasks developed to assess these aspects were used (the Navon Level-Switching task and the Coherent Motion task) [57]. Based on previous research results, we hypothesize that (7) pilots will perform better on generic tasks addressing these aspects compared to non-pilots.

All above-mentioned seven hypotheses were assessed in this study using different measures of gaze behavior, visual scanning and generic skills. Analyzing these hypotheses, will provide us insights in the underlying processes for superior perceptual-cognitive skills in pilots.

## Materials and methods

### Participants

Based on previous research in aviation, we expect high effect sizes for both t-test as well as ANOVA outcomes [40, 51–53]. For a two-way ANOVA [two groups by seven areas of interest (AOI’s)] a total sample size of 32 participants will result in a high statistical power (pwr = .8) for a between group analysis and a sample size of eight participants would result in a high statistical power (p = .8) for both a within and a between interaction analysis. For conducting a t-test analysis, assessing the difference between two independent means, a sample size of 52 participants (26 in each group) is sufficient to obtain high statistical power (pwr = .8).

Taking into account this power analysis, 59 participants were recruited to participate in the study. They were classified into two groups: The first group (non-pilots) consisted of novices with no flight experience (n = 28, mean age 23.86 ± 2.85 years). The second group (pilots) consisted of active airline pilots (n = 30, mean age 26.4 ± 8.22 years) with at least 200 hours of flight experience in flying an Airbus A330-200. The participants were either contacted personally or recruited by the CAE (Canadian Aviation Electronics, academy for pilots located at Steenokkerzeel, Belgium). They all received an e-mail with additional information regarding the protocol and the main focus of the experiment. All participants provided written informed consent and the study was approved by the local University (KU Leuven) ethics committee (G-201504218).

### Equipment and tasks

#### Error-detection task

The task for the participants was to detect a malfunction in one of six cockpit instruments. A total of 16 cockpit video clips were used to produce an error-detection task. Each video clip lasted 33 seconds, showing realistic flight situations (turns, descents and climbs) at an altitude of approximately 1,500–3,000 feet and an airspeed of approximately 150–300 knots from the pilot’s perspective in daylight condition. Video clips were recorded using X-Plane 10 flight simulator (Laminar Research, USA). The instruments panel used was a 2003–2008 era Cirrus instrument panel with the Avidyne Entegral primary flight displays. Out of the 16 clips, 12 clips included a cockpit instrument malfunction in which the instrument froze. Only one instrument malfunctioned per clip and each instrument malfunctioned randomly in two of the 16 clips. An instrument started to malfunction at a random time-point during the video clip. This time-point differed for each malfunction and occurred between the 7^th^ second and the 20^th^ second from the beginning of the clip. In the remaining four clips, the instruments operated flawlessly.

The video clips were presented on a Tobii T120 eye tracking 17-inch monitor (Tobii Technology AB, Sweden) with screen resolution of 1280 x 1024 pixels. This system records eye movements and allows for small head movements (head-movement box: 44 x 22 cm), using the Tobii Studio version 3.2.1 software at a sampling frequency of 60Hz. Raw data (RecordingTimestamp; MediaName; MouseEventX (ADCSpx); MouseEventY (ADCSpx); GazePointLeftX (ADCSpx); GazePointLeftY (ADCSpx); GazePointRightX (ADCSpx); GazePointRightY (ADCSpx); PupilLeft; PupilRight) were extracted from the Tobii Studio version 3.2.1 software and saved. Further processing of the raw data was conducted offline by a Matlab program (co-author I.H.). From the eye tracker signals we calculated fixation locations and durations by the fixation classifier of Hooge and Camps [59]. Based on the fixation durations and locations we performed an area of interest analysis that revealed dwell times, total dwell time, transitions, the transition matrices and entropy measures. Entropy was calculated using the method of Allsop and Gray [37] (for details see: Dependent variables and data processing: Scan entropy).

#### Generic tasks

Coherent motion task: The Coherent Motion task was conducted to assess global motion detection capacity (for a detailed description of the task, see [57]). This test presents 600 moving dots on a computer display for 500 milliseconds. A proportion (5–71%) of the dots in every trial moved in the same direction (i.e., the ‘global motion’). Trials in which only 5% of the moving dots moved in the same direction were classified as the most difficult trials, and trials in which 71% of the moving dots moved in the same direction were classified as the easiest trials. The percentage of moving dots varied randomly between 5 and 71% over the trials, and all participants received the same amount of “difficult” and “easy” trials. Participants were instructed to indicate the global motion by pressing the matching arrow key (i.e., up, down, left or right) on the keyboard after the 500 milliseconds period in which the dots appeared. Since participants could only respond after the moving dots appeared, only accuracy was measured in this task as response time was irrelevant. Each participant performed 20 familiarization trials followed by two sets of 100 trials. An overall accuracy score was calculated as the percentage of correct decisions. The final score was calculated with the following formula after counting the number of correct trials.

Formula:

$$Performance\;in\;the\;Coherent\;Motion\;task\;(\%)=\frac{Number\;of\;correct\;trials}{200}*100$$

Furthermore, to assess the relationship between “difficult” or “easy” trials and the group (pilots versus non-pilots), the sum of correct trials for the 75 most easy and the 75 most difficult trials was calculated separately.

Switching task: The Navon Level-Switching task was conducted in order to measure the ability to selectively attend to and switch between global and local levels of hierarchical visual stimuli (for a detailed description of the task, see [57]). On each trial, a large shape (global level) made up of 18 smaller shapes (local level) appeared. The participants were asked to indicate squares (pressing the F key) or circles (pressing the J key) as fast and accurately as possible. These squares or circles were present either at the global or local level. The participants were not cued about whether they should search for the information at global or local level. They had to check both levels as quickly as possible. Thirty-two trial pairs were presented at random. One trial pair consists of two trials in which the circle or square had to be detected in one trial either at a global (G) or a local (L) level so possible pair combinations were: GL, LG, GG, LL. Response times could be influenced by the type of trial pair that occurred, especially when a switch between local and global detection or vice versa was necessary. Mean global-local and local-global reaction time costs were computed based on the subtractions of local from global (GL pair) and global from local (LG pair) trial reaction times. During the Navon Level-Switching task, accuracy as well as response time were measured. Accuracy was calculated separately for global and local detection, using the following formula:

$$Accuracy\;in\;detecting\;global\;figures\;(\%)=\frac{Number\;of\;correct\;global\;detections}{32}*100$$

$$Accuracy\;in\;detecting\;local\;figures\;(\%)\;=\;\frac{Number\;of\;correct\;local\;detections}{32}*100$$

Response time was measured and used as indication of fluency in attentional switch. For this analysis, the difference in response time to detected a global figure followed by a local figure and the vice-versa were calculated and indicated the time cost to switch attention from global to local detection and from local to global detection.

During each generic task feedback was given by a green cross for correct responses or a red cross for incorrect responses.

### Procedure

For reproducibility, the protocol is available on protocols.io; DOI number: dx.doi.org/10.17504/protocols.io.sk3ecyn. The study consisted of two parts: (1) error-detection cockpit task and (2) generic tasks. Before and after the test session, participants filled out a questionnaire regarding their Rate of Perceived Exertion (RPE) [50]. Originally the RPE scale was used to rate physical exertion, with associated increases in haert rate. The RPE values ranged from 6 to 20 and these ratings were originally used to denote heart rates ranging from 60 to 200 beats per minute. However, other factors (e.g., lack of sleep, performance of difficult cognitive task) can also influence RPE values [50].

#### Error-detection cockpit task

Before starting the error-detection task and in order to familiarize the participants with the glass cockpit and the instruments, each participant received a 10-minute instructional clip about the positions and functioning of the different instruments in the glass cockpit. Thereafter, the participants were seated at a distance of 60 cm from the Tobii monitor and a nine-point gaze calibration was performed. The participants were instructed to hold their head steady throughout the whole task. Then, the participants had to complete the 16-clips error-detection cockpit task. The instructional clip and the task clips were created by a former pilot with over 1,000 hours of flight experience (co-author G.Z.). Participants were instructed to monitor the six main instruments in the cockpit (i.e., airspeed, attitude, altitude, turn, heading, and vertical speed). When a malfunction occurred, it was the participants’ task to identify it as fast and as accurately as possible by clicking on the failing instrument with the mouse. Each clip started with a three second announcement “Get ready”, followed by 30 seconds of flight maneuvers.

After each clip, the participants were asked to respond to the multiple-choice question: ‘I did not click because:’. The possible answers were: Because ‘there was no error’, because ‘I was too late’, because ‘I have no idea’ or ‘irrelevant’. In order to keep the trials consistent, the multiple-choice question was presented after every trial, even when the participant clicked on a failing instrument during the task. If a participant did not identify a malfunction and therefore did not click, the question allowed him to explain why. Participants who were too late to click (they noticed a malfunction but were not able to click before the 33-second clip ended) were asked which instrument did not function correctly and this answer was written down. The entire session lasted approximately 40 minutes. No feedback was given to the participants and gaze behavior was recorded throughout the task.

#### Generic tasks

The next day, the second part of the experiment took place during which the two generic tasks were completed. Both tasks were completed on a laptop computer and every participant started with the Coherent Motion task followed by the Navon Level-Switching task. Each task took approximately 15 minutes. No eye-tracking was conducted during the generic tasks.

### Dependent variables and data processing

All data available on OSF, reference number: osf.io/3hsxu.

#### Decision-making

Accuracy values in the error-detection task were calculated by giving the score of one for correct answers and zero for incorrect answers. A score of one was also given when a participant indicated ‘There was no error’ correctly or indicated verbally the correct malfunctioning instrument after replying ‘I was too late’ in the multiple-choice question that appeared after each trial. In addition, the participants were allowed to click on as many instruments as they wanted so they could change their answers constantly during the 33-seconds video clips. However, an appropriate adjustment for the number of attempts was made afterwards (score 1 if trial was completed correctly, score 1 divided by the number of prior attempts if trial was completed with several mouse clicks). Detection time was calculated as the time between the presentation of the malfunction and its identification with a mouse click. In case of multiple mouse clicks, the detection time was calculated as time between the presentation of the malfunction and the first correct mouse click. Since late but correct responses were very rare, only mouse clicks were used to calculate detection times. Lastly, performance index (PI) was calculated using both accuracy and detection time using the following formula:

$$PI=\frac{ACC}{DT}*100;$$

where ACC is the accuracy score and DT is the mean detection time over all trials of one participant [60].

#### Eye-tracking data

All eye-tracking data were assessed from the beginning of the video until the participant’s response. This was done as most participants, especially novices, tended to relax and stopped monitoring the video after the mouse click. Data recording prior to mouse-clicks lasted between 9.58 and 33 seconds (mean 27.61 ± 6.39 seconds) and the average length of data that has been analyzed was similar for all groups.

#### Visual search behavior

**Fig 1:**
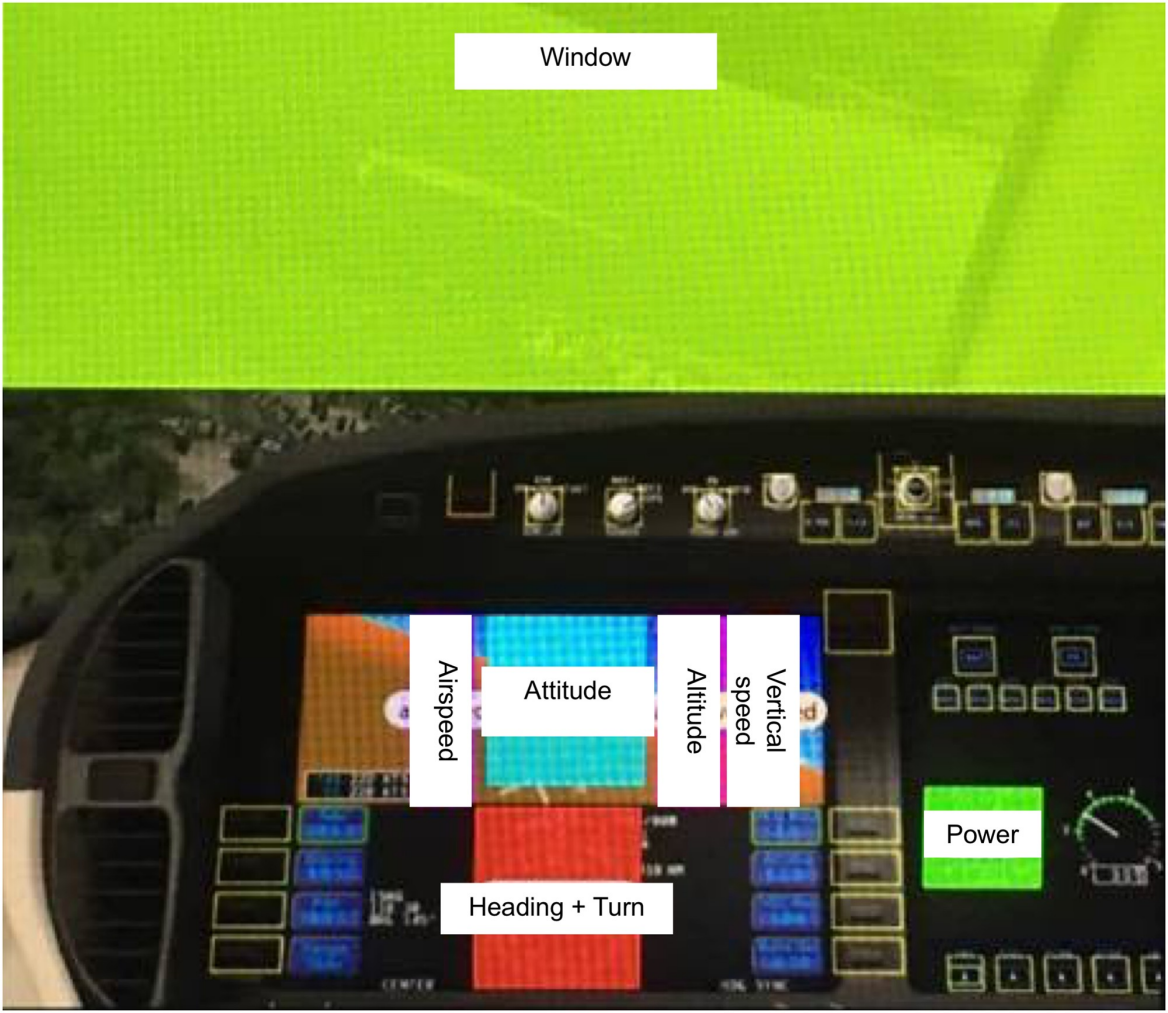
Overview of the different AOI’s: Window (green), Airspeed (purple), Attitude (blue), Altitude (pink), Vertical speed (orange), Heading + turn (red) and Power (green in right corner).

Seven Areas of Interest (AOI) were defined: six instruments within the digital display (Airspeed (60 X 160 pixels); Attitude (134 X 160 pixels); Altitude (66 X 160 pixels); Vertical Speed (62 X 160 pixels); Heading + Turn (146 X 154 pixels); Power (200 X 98 pixels)) and the outside window (936 X 502 pixels) (Fig 1). Dwell times, number of dwells, number of transitions and number of visited areas of interest (AOI) were assessed to quantify the “visual search behavior”. In line with Holmqvist et al. (2011), a dwell was defined as one visit in an AOI, from entry to exit and returns to the AOI are counted as new dwells. Dwell time is then the duration gaze remained inside the AOI measured from entry to exit. The total dwell time was characterized as the sum of all dwell times in one AOI. The number of dwells was defined as the number of visits to an AOI. The average dwell time was calculated as the average of all the dwell times in a certain AOI during the task. Finally, a transition represented a gaze shift from one AOI to another. Furthermore, the participant’s visual coverage was assessed based on the average number of transitions between extreme AOI’s (Airspeed—Vertical Speed; Airspeed—Heading + Turn; Vertical Speed—Heading + Turn; Airspeed—Power), which provides further insights in the extension of the visual span. Extreme AOI’s were defined as AOI’s located across from each other at opposite sides of the instrument panel. A saccade between these AOI’s suggests that other AOI’s located in between the two sides were overlooked. Lastly, the average dwell time outside the different AOI’s (NOT-area) was also assessed as this measure could be seen as being an indicator for parafoveally information processing, but further research is essential to assure this [56, 61–63]. Also, another measure–the time between the occurrence of the error and the first dwell on this failing instrument–was used to further support the hypothesis that experts might process information parafoveally. These assumptions conserning parafoveal information processing rely on previous research in chess in which more fixations of longer durations between the chess pieces were observed in experts while more fixations on the chess pieces themselves were observed in novices. These results suggest an extended visual span in experts [62, 63]. The extended visual span of experts would also mean that while examining structured, but not random, chess configurations, experts would make greater use of parafoveal processing to extract information from a larger portion of a chess board during one fixation [62, 63].

#### Scan entropy

To assess the systematicity of the scan patterns, an entropy analysis was conducted [37] using the formula:

$$\text{Entropy}=\sum_{\text{i}=1}^{\text{n}}\text{p}\left(\text{i}\right)\left[\sum_{\text{j}=1}^{\text{n}}\;\text{p}\;\left(\frac{\text{j}}{\text{i}}\right){\;\text{log}}_{2}\;\text{p}\;\left(\frac{\text{j}}{\text{i}}\right)\right]\;,\text{i}\neq \text{j}$$

For this analysis, first-order transition matrices were set-up, characterizing the transitions between different areas of interest [p(i to j), where i represents the ‘from’ AOI and j represents the ‘to’ AOI]. Separate matrices were calculated for each participant and for all trials. The separate transition frequency matrices were converted into conditional transition-probability matrices of p(j|i), which gives a 1^st^ order Markov process where the probability of fixating on the j^th^ AOI is based on the current dwell on the i^th^ AOI. When applied to the conditional transition-probability matrices, entropy indicates the randomness, or alternatively the predictability, of a participant’s scan behavior. This measure is therefore highly applicable for identifying differences in scanning behavior between participants with different levels of expertise or performance. The observed entropy of the matrices was calculated using an adaptation [64] of Brillouin’s conditional information equation [65]: where p(i) is the zero-order probability of fixating upon the i^th^ AOI based on the number of dwells on this area, p(j|i) is the conditional probability of viewing AOI j based on a current dwell on AOI i, and n is the number of AOIs [37].

Since eye-tracking data was only assessed until the detection time-point, the minimal number of transitions required for a stable entropy value was calculated. In a simulation, the number of random transitions between all seven AOI’s was varied to compute the entropy value. When the number of transitions was higher than 20, the entropy value became stable as function of the number of transitions. Hence, entropy values related to trials with less than 20 transitions were excluded. This analysis assured that the entropy value was independent of the time over which eye-tracking data was analyzed.

### Data analysis

All dependent variables were analyzed for expertise (pilots versus non-pilots) and for performance (best eight versus worst eight performers in each group). To analyze accuracy in the error-detection task, an independent t-test analysis was conducted to study group differences between pilots and non-pilots. Furthermore, a two-way analysis of variance (ANOVA, Group X AOI) with repeated measures on the AOI factor was used to analyze the average dwell time and number of dwells on each AOI to gain insights in the visual search behavior of both expertise as well as performance groups. The other dependent variables were analyzed using independent t-tests to further analyze gaze behavior differences between expertise and performance groups [number of transitions, average number of AOI’s visited in one trial, number of transitions between extreme AOI’s (see above) and average time between the occurrence of the error and the first dwell on the failing instrument] and scan entropy (average entropy value). Performance scores for the generic tasks, and questionnaires (RPE and confidence level) were assessed using independent t-tests. For the Coherent motion task an analysis of the relationship between task difficulty and group affiliation was also conducted. For this analysis, a two-way ANOVA (Group X Level of difficulty) with repeated measures on the Level of difficulty factor was used. In order to control for multiple comparisons, the false discovery rate (FDR) method was used [66, 67]. Lastly, to analyze the relationship between the performance in the generic tasks and the performance in the error-detection task, a stepwise multiple regression analysis was conducted.

#### Eye-tracking data quality

Data quality was assessed for each trial of each participant. Precision and data loss measures were used to decide which trials and which participants should be excluded. Data loss refers to samples in which the eye tracking device failed to locate the pupil and as a consequence did not record gaze. For precision, the Root Mean Square (RMS) of the Euclidean distances between eye-tracking data samples during periods of eye fixations were used. High RMS values indicate low precision [68]. The average of the RMS values over all trials plus two standard deviations was set as cut-off for selection of trials with low precision. These trials were excluded from final data analysis. Also, data loss was analyzed to exclude trials. A maximum of 20% data loss per trial was set as cut-off for selection of valid trials for inclusion in the final data analysis [68, 69].

#### Other exclusion criteria

Each dependent variable was checked for outliers (higher or lower values than the average over all trials of all participants ± three standard deviations) and these values were deleted.

## Results

### Data exclusion

Following an analysis of data quality (RMS and data loss), data from two non-pilots and six pilots were completely deleted because less than seven repetitions of the task remained after excluding trials with low data quality. Furthermore, 9% of all trials from the included participants were excluded (due to low data quality) from further analysis. After the exclusions, data from 24 pilots and 26 non-pilots were included in the analyses.

### Error-detection accuracy, detection time and performance index

An independent t-test indicated a significantly higher accuracy score for detecting the failing instrument in the pilot group compared to the non-pilot group (t(1,48) = 4.26, p < .001, Cohen’s d = 1.21). Respectively, 2% and 3% of the correct answers were provided too late in the non-pilot and the pilot group. Also, a higher performance index was observed for the pilot group compared to the non-pilot group (t(1,48) = 4.22, p < .001, Cohen’s d = 1.20). The time needed to detect a malfunctioning instrument did not differ significantly between groups (t(1,48) = -1.91, p = .06, Cohen’s d = .55) (Table 1).

**Table 1:** Error-detection accuracy, detection time, and performance index for the cockpit task per group (mean ± SD).

Variable	Pilots	Non-Pilots
Accuracy (%)	64.32 ± 12.77*	48.20 ± 13.92
Detection time (sec)	9.43 ± 1.89	10.69 ± 2.67
Performance index	702.34 ± 165.26*	481.97 ± 200.67

* significant different from non-pilots (p <.001)

### Visual search behavior

#### Dwell time

**Fig 2:**
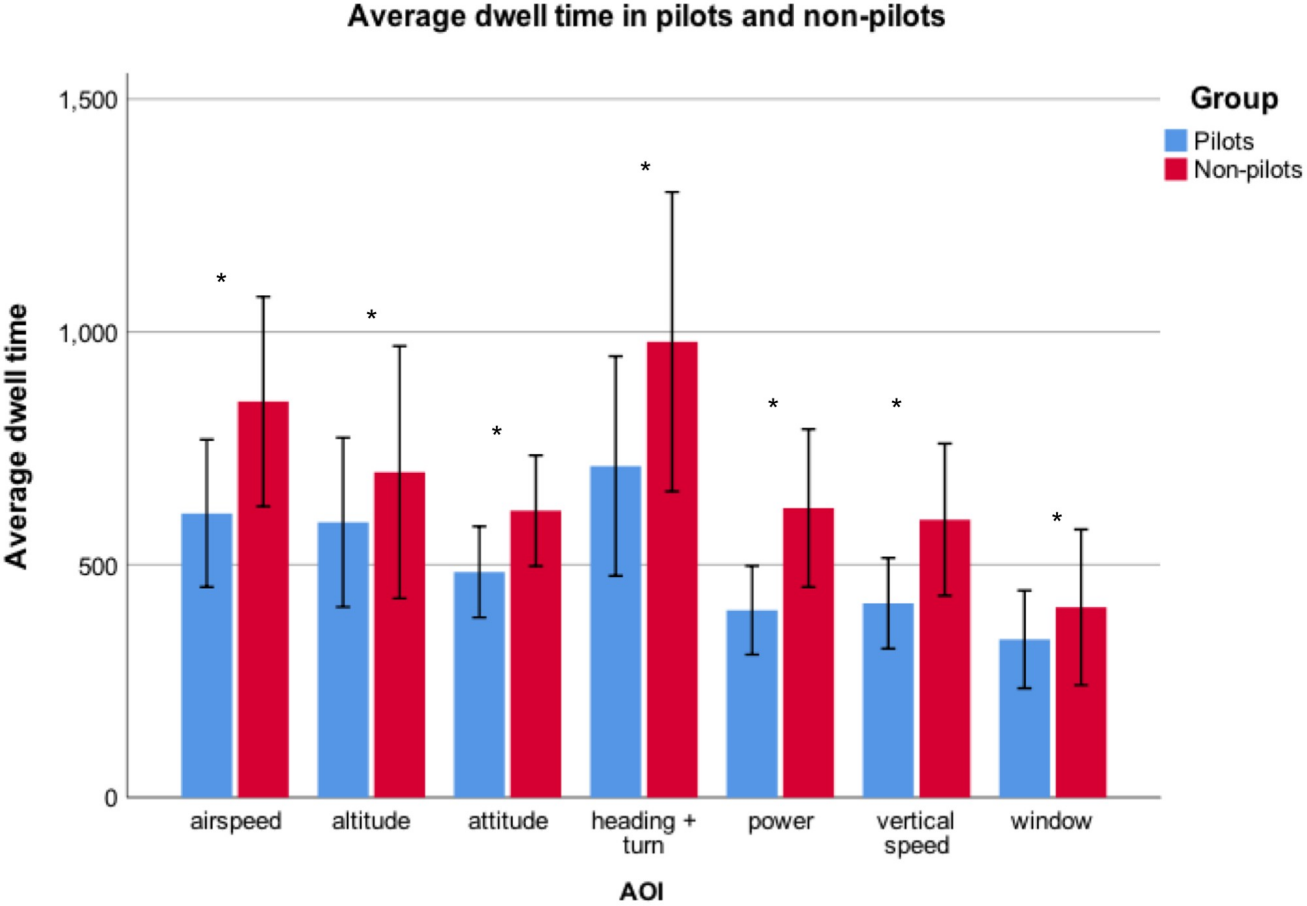
Comparison of mean dwell times on AOI’s between pilots and non-pilots (error bars represent SD and * indicates p < .05).

A two-way repeated measures ANOVA (Group X AOI) on the AOI factor, revealed a main effect for Group (F(1,36) = 34.61, p < .001, partial η^2^ = .49). The average dwell time of the pilots (508.10 ± 210.67 ms) was shorter than that of the non-pilots (681.59 ± 280.04 ms) (Fig 2). A main effect for AOI was also found (F(3.71,133.70) = 31.25, p < .001, partial η^2^ = .47), indicating that, for both groups, longer dwell times were observed on Heading + Turn. No significant interaction was found between Group X AOI (F(3.71,133.70) = 1.70, p = .16, partial η^2^ = .05).

The same analysis was conducted to compare the best eight with the worst eight performing pilots. A main effect for AOI (F(2.11,19.02) = 14.95, p < .001, partial η^2^ = .62) was found. However, no significant effects were observed for group (F(1,9) = .05, p = .82, partial η^2^ = .01) and no significant interaction was found (F(2.11,19.02) = 1.07, p = .37, partial η^2^ = .11).

A similar analysis was conducted for high and low performing non-pilots. A significant main effect was observed for AOI (F(6,72) = 9.38, p < .001, partial η^2^ = .44). No significant effect was observed for Group (F(1,12) = .49, p = .50, partial η^2^ = .04) and no significant interaction was found (F(6,72) = .89, p = .51, partial η^2^ = .07).

Lastly, the dwell times outside the AOI’s (the NOT-area) were assessed using an independent t-test. The results indicate a significant higher average dwell time in the NOT-area for pilots (2,751.63 ± 796.08 ms) compared to non-pilots (2,011.77 ± 944.55 ms) (t(1,48) = 4.51, p < .001, Cohen’s d = 1.27). No differences in dwell times on this area were observed between high and low performing pilots (t(1,14) = .42, p = .68, Cohen’s d = .21) or between high and low performing non-pilots (t(1,14) = .45, p = .66, Cohen’s d = .22).

#### Time to first dwell on the failing instrument

An independent samples t-test indicated that pilots (2,710.05 ± 971.62 ms) had a shorter time to first dwell on the failing instrument after the error occurred compared to non-pilots (3,775.52 ± 1,428.28 ms) (t(1,48) = -3.02, p = .004, Cohen’s d = .87).

#### Number of dwells

**Fig 3:**
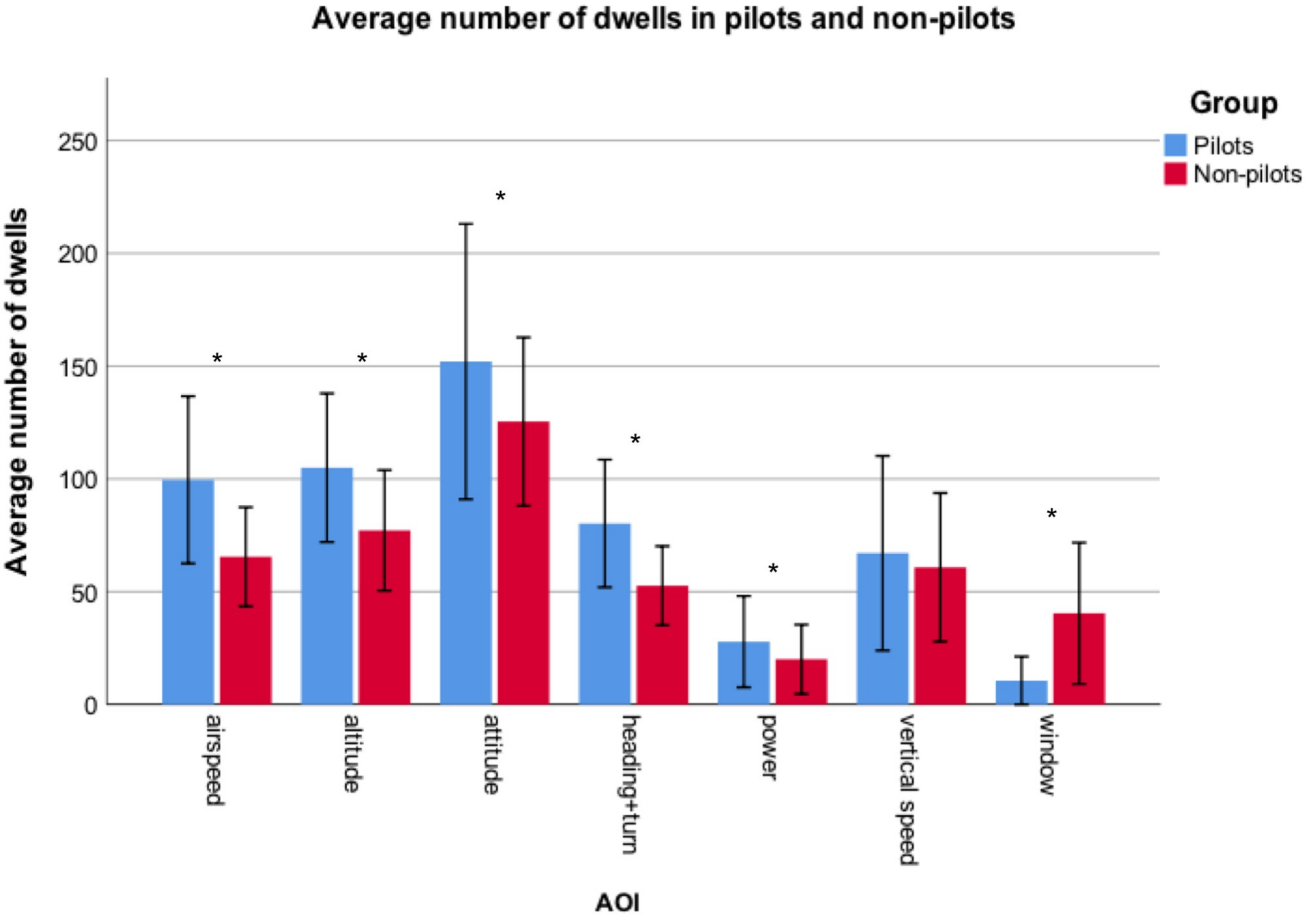
Comparison of the mean number of dwells between pilots and non-pilots (error bars represent SD and * indicates p < .05).

A two-way repeated measures ANOVA (Group X AOI) on the AOI factor, revealed an interaction between Group and AOI (F(3.71,163.43) = 6.27, p < .001, partial η^2^ = .13) for the average number of dwells on each AOI. An examination of the 95% confidence intervals of the number of dwells to each AOI in the pilot and the non-pilot group, revealed that pilots made more dwells to all AOI’s except for Vertical speed and Window. There was no difference in number of dwells to the Vertical speed between pilots and non-pilots, and pilots made less dwells to the Window compared to non-pilots (pilots: 10.54 ± 10.73; non-pilots: 40.41 ± 31.29) (Fig 3). The main effects for Group (F(1,44) = 8.44, p = .006, partial η^2^ = .16) and for AOI (F(3.71,163.43) = 80.21, p < .001, partial η^2^ = .65) were also significant. With highest number of dwells, made by both groups, found for the Attitude indicator). The average number of dwells was significantly different between pilots (77.47 ± 57.12) and non-pilots (63.14 ± 40.35).

The same analysis was conducted to compare the best eight with the worst eight performing pilots. The two-way ANOVA revealed a main Group effect (F(1,14) = 6.00, p = .03, partial η^2^ = .30). The average number of dwells on each AOI differed significantly between high (68.04 ± 45.23) and low performing (84.64 ± 62.38) pilots (p = .03). In addition, a significant AOI effect was observed (F(2.68,37.53) = 30.59, p < .001, partial η^2^ = .69). No significant interaction was observed (F(2.68,37.53) = 1.04, p = .38, partial η^2^ = .07).

Lastly, comparing high and low performing non-pilots revealed only a main effect for AOI (F(3.62,50.64) = 31.49, p < .001, partial η^2^ = .69).

#### Number of visited AOIs per trial

Pilots appeared to visit on average less AOIs per trial (5.67 ± .37) compared to non-pilots (5.93 ± .47) (t(1,48) = -2.18, p = .04, Cohen’s d = .62). No differences in average number of visited AOI’s per trial were found between high and low performing pilots (t(1,14) = -.73, p = .48, Cohen’s d = .36) or between high and low performing non-pilots (t(1,14) = -.89, p = .39, Cohen’s d = .44).

#### Number of transitions

Analysis of the average number of transitions per trial indicated that pilots made significantly more transitions (34.47 ± 5.86) between different AOI’s compared to non-pilots (27.50 ± 5.07) (t(1,48) = 4.51, p < .001, Cohen’s d = 1.27). There was no difference in the average number of transitions per trial between Low performing pilots (35.40 ± 5.44) and high performing pilots (30.48 ± 4.81) (t(1,14) = -1.92, p = .08, Cohen’s d = .96). No significant differences were found between the best eight (26.54 ± 4.6) and the worst eight (30.06 ± 4.88) performing non-pilots (t(1,14) = -1.48, p = .16, Cohen’s d = .74).

#### Number of transitions between extreme AOI’s

Transition matrices indicate the spread of transitions between the different AOI’s.

A comparison of the average transition matrix between pilots and non-pilots revealed a significant wider visual scanning in pilots. Pilots made more transitions between extreme AOI’s (Airspeed—Vertical Speed; Airspeed—Heading + Turn, Vertical Speed—Heading + Turn and Airspeed—Power) (.65 ± .21) compared to non-pilots (.32 ± .10) (t(1,9.96) = 3.89, p = .003, Cohen’s d = 1.94). In addition, a comparison of the best eight and the worst eight performing pilots revealed that high performing pilots appeared to make less transitions between extreme AOI’s (.50 ± .25) compared to low performing pilots (.88 ± .15) (t(1,10) = -3.15, p = .01, Cohen’s d = 1.82). This difference was not observed in low (.46 ± .10) and high performing (.36 ± .16) non-pilots (t(1,8.24) = -1.21, p = .26, Cohen’s d = .70).

### Scan entropy

No significant differences in entropy values were observed between pilots and non-pilots (t(1,48) = 1.48, p = .15, Cohen’s d = .42). In addition, no significant differences in entropy values were observed between high and low performing pilots (t(1,14) = -1.01, p = .33, Cohen’s d = .50) or between high and low performing non-pilots (t(1,14) = -.15, p = .88, Cohen’s d = .07).

### Performance and response time scores in the generic tasks

No significant differences between the two groups were observed in accuracy scores (Coherent Motion task: t(1,46) = .57, p = .57, Cohen’s d = .17; Navon Level-Switching task: t_local_(1,47) = .33, p = .74, Cohen’s d = .09; t_global_(1,37.77) = 1.68, p = .10, Cohen’s d = .48). For the Navon Level-Switching task, no significant difference in time cost to switch from local to global was observed between groups (t(1,47) = .26, p = .80, Cohen’s d = .07). Although, the reverse switch (GL pair) appeared to cost less in pilots compared to non-pilots (t(1,47) = -2.64, p = .01, Cohen’s d = .75); FDR cutoff p-value = .01 (Table 2). Lastly, a two-way ANOVA revealed no significant interaction between group and Coherent motion task difficulty (F(1,47) = .003, p = .953, partial η^2^ = .00).

**Table 2:** Generic task performance in pilots and non-pilots: Coherent motion detection accuracy, local detection accuracy, global detection accuracy, response time difference between local (L) and global (G) detection and vice-versa (mean ±SD).

Coherent motion test		
Variable	Pilots	Non-Pilots
Accuracy (%)	68.60 ± 8.81	66.63 ± 14.47
Navon-level switching task		
Variable		
Accuracy local (%)	90.63 ± 13.13	89.50 ± 10.82
Accuracy global (%)	95.31 ± 4.29	92.07 ± 8.67
Response time LG (sec)	-.02 ± .11	-.03 ± .10
Response time GL (sec)	.04 ± .10*	.11 ± .09

* significant difference from non-pilots (p <.05)

### Relationship between generic task performance and error-detection task performance

A stepwise multiple regression analysis was conducted to assess which independent variables [AccLocal (accuracy in detecting the local figure); AccGlobal (accuracy in detecting the global figure); GL (time cost to switch from global figure detection to local figure detection); LG (time cost to switch from local figure detection to global figure detection) and accuracy score on the Coherent motion task)] could predict the accuracy on the error-detection cockpit task. This regression analysis revealed that GL and AccGlobal predicted 21.4% of the variance in accuracy scores in the specific error-detection cockpit task (p = .008) (Table 3). More specifically, the variation in error-detection accuracy can be calculated with the following equation: Error-detection accuracy = -6.14–64.72 * (GL) + .73 *(AccGlobal).

**Table 3:** Results of the multiple regression analysis.

Step	Variable	R	R Squared	Adjusted R Squared	Significant F Change
1	GL	.347	.120	.099	.023
2	GL+ AccGlobal	.462	.214	.174	.035

Dependent variable = Accuracy. GL = time cost to switch from global figure detection to local figure detection, AccGlobal = accuracy score on global figure detection trials.

### RPE-scale

No significant differences in RPE were observed between pilots and non-pilots (t(1,46) = -1.73, p = .09, Cohen’s d = .50). Although, a trend suggesting that this task was less demanding for the pilots’ group was observed. No significant difference in RPE was observed between high and low performing pilots (t(1,13) = -1.08, p = .30, Cohen’s d = .55) and between high and low performing non-pilots (t(1,9.96) = .20, p = .84, Cohen’s d = .11).

## Discussion

The purpose of this study was to unravel pilots’ gaze behavior and attentional generic skills to bridge the gap in knowledge with respect to underlying processes that are linked to expertise and superior flight performance. For successful completion of a complex task such as flying of an aircraft, pilots are assumed to possess superior perceptual-cognitive skills [70, 71]. Previous research suggested three theories addressing the relations between gaze behavior and expert performance. Namely, (1) the long-term working memory theory [15], (2) the information-reduction hypothesis [16], and (3) the holistic model of image perception [17]. Furthermore, previous results have also shown that low scan entropy values, that are related to a systematic scan pattern, might also be a predictor for expertise and superior perceptual-cognitive skills [36–39, 41–42]. In addition, some generic perceptual-cognitive skills which have been examined through inclusion of two generic tasks (i.e.: the Navon Level-Switching task and the Coherent Motion task [57]) were put forward as possible underlying processes for expert performance. In the present study, seven hypotheses were tested. The results support four of the seven hypotheses: compared to non-pilots, pilots make more transitions and use shorter dwell times (hypothesis 1), pilots make fewer dwells of shorter durations to task-irrelevant areas and more dwells of longer durations to task-relevant areas (hypothesis 2), pilots have a wider visual span (hypothesis 3), and pilots perform better in one of the four performance measures of the Navon Level-Switching task (time cost to switch between local and global information processing) (hypothesis 7). All proposed hypotheses are discussed according to the three theories, scan entropy, and generic skills.

### The three theories of gaze behavior and expert performance in a complex error-detection cockpit task

Based on *the long-term working memory theory* it is expected that (i) pilots used more transitions and shorter dwell times [15]. Our observations support this theory, because pilots performed better and make more transitions of shorter durations between the instruments compared to non-pilots. This suggests that pilots were able to capture the information needed to complete the task in less time. These observations are in line with previous research results addressing differences in gaze behavior between different expertise levels and provides supporting evidence for the long-term working memory theory [15, 18, 25, 29, 38, 72]. Furthermore, our results are in line with previous study results [73] suggesting that the dwell time on instruments is shorter during a monitoring cockpit task in pilots.

The relationship between gaze behavior and expert performance can also be explained by *the information-reduction hypothesis* [16]. According to this theory, it is expected that (ii) pilots made more dwells to the instruments in the cockpit and less to the window. Once again, the results of the current study support this theory: compared to non-pilots, pilots had shorter dwell times on the task-irrelevant area (i.e. Window) and visited this area less often. More specifically, pilots visited the Window approximately four times less compared to non-pilots. Also, a second analysis was conducted to further validate the information-reduction hypothesis with the obtained eye-tracking data by examining the group difference in the average number of AOIs visited per trial. Results showed that pilots visited on average significantly less AOI’s per clip, compared to non-pilots. One possible explanation for these results might be that fixating on areas between the instruments and on the window interferes with task performance, but this should be studied further.

In summary, our observations clearly indicate that pilots avoided task-irrelevant areas and spent more time dwelling on task-relevant areas compared to non-pilots. This observation is in line with previous research results, implying that less experienced pilots fixated more on the Window compared to experts and that experts in general fixated less and shorter on this area compared to the cockpit panel instruments [7, 74–76].

According to *the holistic model of image perception* [17], it is expected that; (iii) pilots used longer dwell times on the areas between the instruments in the cockpit (NOT-area), and a shorter time to first dwell on the failing instrument after the error occurred, which might suggest that they were relying more on peripheral information processing; this observation was also put forward in the research results of this study. The finding that experts were better at identifying a failing instrument while they fixated more on the area next to the instruments, suggests that pilots might have used their peripheral vision and thereby have an enlarged visual span for information processing [17]. Furthermore, pilots dwelled faster on the failing instrument after the error occurred. This can be explained by the global-local information processing as proposed by the holistic model of image processing [17]. It is possible that due to their extended visual span, pilots captured the whole scene during a global scan, followed by a local scan that was used to analyze possible abnormalities in more detail. Moreover, the shorter dwell times and a higher number of dwells found in pilots, can also be explained by the global-local information processing strategy used by experts. Based on this strategy, pilots should be able to detect a malfunction in the first stage of search, possibly by using their peripheral vision. The use of peripheral vision is expected to provide a global scan of the scene and thereby requires only a short dwell on the target AOI for confirmation. During the second stage, a cross-referencing for other potential malfunctions is expected, during which more dwells or fixations will be used. During this second phase a more local scan will be used [26, 29, 30, 77–80]. Again, these observations are in line with research results which explored gaze behavior characteristics in relation to expertise in other expert domains; such as, in chess and medicine [24–26, 29, 62, 63, 77–80]. In aviation, only a few of the studies examined the effects of peripheral vision on performance. A previous study examined flight performance in highly experienced and less experienced pilots when peripheral vision was blocked in order to assess the pilots’ peripheral vision information processing capacity [81]. To measure peripheral information processing, only the fixated instrument was visible, while the others were covered. The performance of all pilots decreased when peripheral vision was blocked, with the highly experienced pilots’ performance suffering more than that of the less experienced pilots.

The results of the current study suggest that the underlying processes required to obtain superior performance in a specific error-detection cockpit task, as described by the three theories, mostly evolve with experience. However, comparison of gaze behavior between the eight bests and eight worst performers (both from the pilot group as well as from the non-pilot group) revealed some differences between pilots but not between non-pilots. As mentioned before, pilots made more transitions between instruments, compared to non-pilots and the spread of these transitions differed between high and low performing pilots. Surprisingly, high performing pilots showed less transitions between extreme AOI’s compared to low performing pilots. In contrast to the holistic model of image perception, the most extended visual span was not the most beneficial for successful task performance. Since automatization of processes, like visual scanning, evolves with experience, it can be assumed that the worst performing pilots developed a less efficient scanning behavior. During inefficient scanning, it is assumed that the pilots’ eyes move too fast over the cockpit instrument panel and miss essential information. This is in line with previous research results indicating that skill-based errors are the main cause for aviation accidents [82]. Furthermore, the results of our study are similar to previous study results [70], showing that a shorter total scan length leads to better flight performance.

Finally, we found that number of dwells on the different instruments did not only differ between pilots and non-pilots, but also between high and low performing pilots. This observation contradicted the hypothesis suggesting that high performing participants (irrespective of their level of expertise) were expected to adapt an expert-like gaze behavior. Regarding the observations of the current study, pilots showed more dwells of shorter durations on cockpit instruments, more transitions between instruments, and less dwells on the irrelevant area (i.e., Window) compared to non-pilots. Surprisingly, high performing pilots showed less dwells compared to low performing pilots. However, this effect was not observed between high and low performing non-pilots. The above-mentioned observation might suggest that low performing pilots used a more exhaustive search strategy, compared to high performing pilots, which is supposed to be less efficient [18,83]. This is in line with the statement made by Rayner that “The number of fixations made can be used to indicate efficiency of search with the number of fixations overall negatively correlated with search efficiency” [84]. It can be assumed that the same statement is also applicable for the number of dwells.

### Analysis of the relation between scan entropy and task performance

The results of the entropy analyses did not support our hypotheses that pilots’ and/or high-performers’ scan pattern will be more systematic, resulting in a lower scan entropy. Specifically, our observations revealed no significant changes in the levels of scan entropy between pilots and non-pilots. Furthermore, no significant associations were found between entropy measurers and the level of expertise or the level of performance. In contrast to the results of the current study, previous study results showed relations between a lower scan entropy and expertise as well as performance during different flight tasks [37, 40]. The study of Kasarskis et al. reported lower scan entropy values in expert pilots as compared to non-expert pilots during a landing task [40]. In another study, the level of scan entropy increased with the induction of anxiety [37], showing that higher anxiety state and higher levels of scan entropy in the cockpit were negatively related with performance.

Taken together, it appears that the results for scan entropy obtained in the current study were in contradiction with previous results. There are few explanations for this discrepancy. First, previous studies [37, 40] were conducted using a cockpit display with analog instruments, whereas in the present study a digital display of cockpit instruments was used. Taking this difference into account, we propose that analog displays might provide a better opportunity for participants to use a more structured scan since instruments in the analog cockpit setting are located in a distinctly different location. In contrast, in the current study we used a digital cockpit setting with instruments mostly located on one large primary display. In this display, instruments are organized in a smaller visual field, hence para-foveal vision can extract more information, which might reduce the need for systematic scanning in experts.

Also, the task in our study (monitoring) differed from the tasks in these two studies, which reported differences in scanning entropy between experts and novices [39] or between different states of anxiety [37]. Previous research results already provided evidence for the effects of different task instructions on gaze and scanning behavior, both in pilots as well as in other domains [30, 61, 85–89].

### Analysis of the relation between workload, generic attentional skills, and expert performance

In the current study, while no differences were observed in capability to capture essential information from multiple inputs (Coherent Motion task) between pilots and non-pilots, the attentional skills (Navon Level-Switching task) were significantly better in pilots compared to non-pilots [57]. As expected, pilots were more fluent in switching their attention between global and local information processing, suggesting they use a global-local information processing strategy, in line with the holistic model of image processing [17, 38]. These results partially support our hypothesis that pilots will perform better on generic tasks that address extraordinary attentional skills, and the capability to capture essential information from multiple inputs, compared to non-pilots.

Previous research also reported relationships between domain-specific and domain-generic skills [3, 49, 55]. In contrast, other research results reported no relation between domain-generic and domain-specific skills [51, 58]. These reports indicated that there is still much uncertainty in the research domain assessing these relations. To the best of our knowledge, this was never studied in aviation. Monitoring cockpit instruments in general and detection of a cockpit instrument malfunction in particular are expected to require large attentional demands from unexperienced pilots or novices. Therefore, it was predicted that pilots will show superior performance on both generic tasks. In contradiction to this expectation, no effects of expertise on the Coherent Motion task were observed. While this observation was largely in line with the results of a previous publication from our group [58], it was rather a surprising observation in the context of the current study because being able to capture essential information from multiple inputs, as measured with the Coherent Motion task, is one of the most essential skills in aviation. Also, in our study, a monitoring task was used while in the previous study from our group a decision-making task was used. Type of task and instruction can influence research outcomes in different ways. For this reason, it was still interesting to assess the effects of this task in the cockpit setting. Since no effects were observed for the Coherent Motion task, in our research project as well as in the previous project conducted in our group, it can be assumed that the capability to capture essential information from multiple inputs is a skill that only evolves with expertise in domain-specific settings. Besides a relation between attentional generic skills and expertise, also a relation between attentional generic skills and performance was observed. A stepwise regression analysis revealed a relation between Navon Level-Switching task performances and error-detection cockpit task performance. More specifically, the capability to switch attention fluently and process global information was related to the accuracy level in the specific cockpit task. Also, in this specific cockpit task it was important to maintain high global information processing capabilities, since it was not important to read the specific number on the cockpit instruments, but only detect an instrument that has stopped working. Therefore, capturing a global image of the cockpit might provide an advantage for successful cockpit task completion.

In contrast to our hypothesis, the workload of the specific error-detection cockpit task did not differ significantly between pilots and non-pilots. A possible explanation for this is the low difference in attentional generic skills between pilots and non-pilots (only one of the five measures for attentional generic skills showed a significant difference between pilots and non-pilots). According to a previous study, attentional generic skills are expected to be related to task workload [45]. Hence, the low differences in attentional generic skills may well be related to low group differences in task workload.

The obtained results in this study provide insights in the underlying processes that explain expert perceptual-cognitive skills in aviation. Since differences in gaze behavior were observed between high and low performing pilots, it can be assumed that there is an inefficient manner of visual search. Integrating this information in pilot training interventions may reduce the amount of serious accidents in aviation, caused by human error and specifically deficiencies in visual attention.

## Conclusion

In this study, differences in gaze behavior and visual scanning were analyzed between both pilots versus non-pilots, as well as between high versus low performers. Differences in gaze behavior between pilots and non-pilots were in line with three theories that could explain the underlying process for perceptual-cognitive skills: (i) the long-term working memory theory [15], (ii) the information-reduction hypothesis [16] and (iii) the holistic model of image perception [17]. This indicates that in aviation, all three theories capture complementary aspects of expert perceptual-cognitive skilled performance. Mostly, no differences in gaze behavior were observed after conducting the same analysis within the same expertise group, comparing high and low performers, suggesting that the underlying processes for the development of perceptual-cognitive skills mostly evolve with experience. However, the number of dwells differed between high and low performing pilots, indicating that low performing pilots used a more exhaustive search. Also, for the number of transitions between extreme AOI’s, which indicates the width of the visual span, a significant difference was observed between high and low performing pilots. Low performing pilots appeared to make more transitions between extreme AOI’s and thereby use a wider visual span. Our result supports previous research, indicating that most of the aviation accidents are caused by skill-based errors, of which an example is the usage of an inefficient scanning. Thus, scanning with a too wide visual span, can be classified as inefficient scanning and one of the underlying causes for aviation accidents [82].

With respect to generic skills, the results indicate that pilots are better at switching their attention between global and local information processing. Performance on the Navon Level-Switching task was also positively related to the accuracy score on the error-detection cockpit task. Furthermore, these observations might provide further evidence for the holistic model of image processing, since this theory argues for a global-local scanning strategy for information collection.

In summary, the current project provided clear insights in the underlying processes that can explain perceptual-cognitive expertise both using eye-tracking measures as well as generic task performance measures. Furthermore, several gaze characteristics differed between high and low performing pilots, but also some generic task performance outcomes were related to specific error-detection cockpit task accuracy. This observation suggests that there are also certain underlying processes that can predict safe flight performance, which indicates an interesting measure for future screening or training protocols in aviation.
